# Phosphodiesterase 3/4 Inhibitor Zardaverine Exhibits Potent and Selective Antitumor Activity against Hepatocellular Carcinoma Both *In Vitro* and *In Vivo* Independently of Phosphodiesterase Inhibition

**DOI:** 10.1371/journal.pone.0090627

**Published:** 2014-03-05

**Authors:** Liping Sun, Haitian Quan, Chengying Xie, Lei Wang, Youhong Hu, Liguang Lou

**Affiliations:** Shanghai Institute of Materia Medica, Chinese Academy of Sciences, Shanghai, China; Virginia Commonwealth University, United States of America

## Abstract

Hepatocellular carcinoma (HCC) is the fifth common malignancy worldwide and the third leading cause of cancer-related death. Targeted therapies for HCC are being extensively developed with the limited success of sorafinib. In the present study, we investigated the potential antitumor activity of zardaverine, a dual-selective phosphodiesterase (PDE) 3/4 inhibitor in HCC cells both *in vitro* and *in vivo*. Although all zardaverine, PDE3 inhibitor trequinsin and PDE4 inhibitor rolipram increased intracellular cAMP levels through inhibiting PDE activity, only zardaverine significantly and selectively inhibited the proliferation of certain HCC cells, indicating that the antitumor activity of zardaverine is independent of PDE3/4 inhibition and intracellular cAMP levels. Further studies demonstrated that zardaverine induced G_0_/G_1_ phase cell cycle arrest of sensitive HCC cells through dysregulating cell cycle-associated proteins, including Cdk4, Cdk6, Cdk2, Cyclin A, Cyclin E, p21 and Rb. Notably, Rb expression was reversely related to the cell sensitivity to zardaverine. The present findings indicate that zardaverine may have potential as targeted therapies for some HCC, and the likely mechanism of action underlying its selective antitumor activity may be related to its regulation of Rb or Rb-associated signaling in cell cycles.

## Introduction

Hepatocellular carcinoma (HCC) is the fifth common malignancy worldwide and the third leading cause of cancer-related death [1]. Currently, targeted therapies for HCC are being extensively developed with the limited success of sorafinib [2], [3]. This is largely caused by the heterogeneity in HCC and no dominant signaling pathway has been recognized so far [4]. Therefore, the discovery and development of novel agents targeting HCC is of utmost importance.

Cyclic AMP (cAMP), one of the most important intracellular second messengers, which controls a diverse range of cellular processes, exhibits absolutely contrary effects on cell proliferation depending on the cell types. cAMP acts as a positive intracellular signal for cell proliferation in parotid cells, thyroid cells and Swiss 3T3 cells [5]. In contrast, cAMP inhibits the proliferation of malignant gliomas, vascular smooth muscle cells, HEK293 cells, T lymophocytes and HCC cells [6], [7], [8], [9], [10]. A previous report from our laboratory has demonstrated that cAMP suppresses the proliferation of HCC cell line Bel-7402 by inhibiting Akt activation [11]. The level of intracellular cAMP is regulated by a plasma-membrane-bound enzyme adenylyl cyclase that synthesizes cAMP from ATP and cAMP phosphodiesterases that hydrolyze cAMP to adenosine 5′-monophosphate (5′-AMP).

Cyclic nucleotide phosphodiesterases (PDEs) play key roles in cell signaling by hydrolyzing cAMP and cGMP. The PDE superfamily is subgrouped into 11 gene families (*PDE1* to *PDE11*) that include at least 20 different genes to produce more than 50 PDE proteins in mammalian cells [12]. Among these PDE gene families, *PDE4*, *PDE7* and *PDE8* are specific for cAMP [13], [14]. Many tumor cells display significantly decreased cAMP levels because of overexpression of PDEs. It has been reported that cell lines originating from the central nervous system (CNS), lung, breast and melanomas exhibit high activity of PDEs and that a survey of the NCI panel of 60 human tumor cell lines indicate that the major activity of PDEs results from PDE4 [15]. And the selective PDE4 inhibitor rolipram is well investigated as an antitumor agent. It has been indicated that rolipram inhibits the growth of several breast and lung carcinoma cell lines [16], [17], slows the intracranial growth of glioblastoma and medulloblastoma xenografts [18], causes profound growth arrest of chemoresistant KM12C colon cancer cells through combination with low doses of the adenylyl cyclase activator forskolin [19], induces growth suppression and apoptosis in human acute lymphoblastic leukemia (ALL) cells [20], induces chronic lymphocytic leukemia (CLL) apoptosis and could partially overcome the ‘rolipram-resistance’ of CLL cells when combined with the selective PDE3 inhibitor cilostamide [21].

Zardaverine, a dual-selective PDE3/4 inhibitor, was synthesized in 1984 and developed as a potential therapeutic agent for asthma, which inhibits the bronchoconstriction 100-fold more potently than theophylline but with similar efficacy. However, due to its fast elimination, the development of zardaverine was terminated in 1991 [22]. It has been reported that zardaverine is a more potent inhibitor of human peripheral blood mononuclear cells (PBMC) and T-cell proliferation than rolipram [23], [24]. However, whether zardaverine has antitumor activity on solid tumor and, if so, whether through PDE3/4 inhibition, is unknown.

In this report, we investigated the antitumor activity of zardaverine and found that zardaverine displayed potent and selective antitumor activity against HCC both *in vitro* and *in vivo*. More importantly, the antitumor activity of zardaverine is independent of the PDE3/4 inhibition. Our data indicate that zardaverine has potential for the treatment of tumors, especially for HCC.

## Materials and Methods

### Materials

Zardaverine was synthesized at the Shanghai Institute of Materia Medica, Chinese Academy of Sciences, China. Vinorelbine was obtained from Jiangsu Hansoh Pharmaceutical Co. (Lianyungang, China). Sulforhodamine B, propidium iodide (PI), trequinsin hydrochloride, rolipram, amrinone, 8-Br-cAMP, forskolin, paclitaxel were purchased from Sigma–Aldrich (St. Louis, MO, USA).

Antibodies to caspase-8, caspase-9, poly-(ADP-ribose) polymerase (PARP), p21^Waf1/Cip1^, Cdk2, Cdk4, Cdk6, Cyclin D1, Rb, phospho-Rb (Ser780) and GAPDH were purchased from Cell Signaling (Beverly, MA, USA). Antibodies specific for p16, Cyclin A, Cyclin E and caspase-3 were purchased from Santa Cruz Biotechnology (Santa Cruz, CA, USA).

### Cell culture

The human hepatocellular carcinoma cell lines Bel-7402, Bel-7404, SMMC-7721 and QGY-7701 were obtained from the Cell Bank of the Shanghai Institute for Biological Sciences, Chinese Academy of Science (Shanghai, China). The cell lines Hep G_2_, Hep 3B, SNU-387, SNU-398, SNU-423, SNU-475, SK-OV-3, NCI-H460, LNCaP, MDA-MB-231, NCI-N87, U-87 MG, A431 and HCT 116 were purchased from the American Type Culture Collection (Manassas, VA, USA). SNU-739 was purchased from the Korean Cell Line Bank (Seoul, Korea). Bel-7402, Bel-7404, SMMC-7721 and QGY-7701 cells were cultured in RPMI 1640 medium and SNU-739 cells were cultured in Dulbecco’s modified Eagle’s medium supplemented with 10% fetal bovine serum at 37°C in an atmosphere of 5% CO_2_; all other cell lines were cultured according to instructions provided by the ATCC.

### Cell proliferation assay

Cell growth inhibition was determined by sulforhodamine B (SRB) assay as described previously [25]. Briefly, approximately 24 h after plating, cells were incubated with various concentrations of compounds for 72 h followed by SRB assay.

### Western blotting

Western blotting was performed as described previously [25]. Briefly, after drug treatment cells were washed and lysed to obtain cell lysates which were separated by SDS-PAGE and transferred to PVDF membranes followed by blocking, incubation with primary antibody and secondary antibody step by step. Finally, immunoreactive proteins were visualized using the enhanced chemiluminescence system from Thermo Fisher Scientific (Waltham, MA, USA).

### Cell-cycle analysis

Cell cycle distribution was measured using a FACscan flow cytometer (BD Biosciences, San Jose, CA, USA) and analyzed with CellQuest and ModFit LT3.0 software. The detailed experimental procedure was referred to the previous report [25].

### cAMP measurement

Intracellular cAMP levels were measured by a competitive immunoassay based on time-resolved fluorescence resonance energy transfer (TR-FRET) between the fluorescent reporters cAMP-d2 and anti-cAMP-cryptate using a cAMP dynamic 2 kit (Cisbio Bioassays, France). Cells were cultured in 96-well microplates with a concentration at 2×10^4^ cells/well overnight at 37°C. Different concentrations of compounds were added to cell culture and incubated for appropriate periods at 37°C. Next, the medium was removed and lysis buffer (50 µl/well) supplied in the kit was added to lyse cells. Then, 10 µl cell lysate/well was transferred to 384-well microplates followed by the addition of cAMP-d2 and anti-cAMP-cryptate (5 µl each). Each treatment was conducted as triplicate to account for variability. Incubation for 1 h at room temperature was followed by a 5 min centrifugation at 300 × g. The plates were read out with Synergy H4 Hybrid Multi-Mode Microplate Reader (BioTek Instruments, Inc. Winooski, VT, USA). Data processing and quantification of cAMP were performed according to the manufacturer’s instructions.

cAMP levels of tumors were determined as described above. 100 mg of tumor tissue was lysed in 200 µl RIPA (20 mM Tris-HCl (pH 7.5), 150 mM NaCl, 1 mM Na_2_EDTA, 1 mM EGTA, 1% NP-40, 1% sodium deoxycholate, 1 mM Na_3_VO_4_) to obtain homogenate. After centrifugation at 10000 × g for 15 min, the supernatant was used to test the cAMP concentration.

### *In vivo* study

Female nude mice (Balb/cA-nude, 5–6 wk old) were purchased from Shanghai Laboratory Animal Center, Chinese Academy of Sciences (Shanghai, China). Human tumor xenografts of Bel-7402 and HCT 116 cells were established by subcutaneously inoculating cells into nude mice. When tumor volumes reached 100–200 mm^3^, mice were randomly assigned to control and treatment groups and treated with vehicle, zardaverine (p.o.) or CPT-11 (i.p.) respectively. Tumor volume was calculated as (length×width^2^)/2. The use of animals was approved by the Institute animal reviews boards of Shanghai Institute of Materia Medica, Chinese Academy of Sciences, with confirm adherence to the ethical guidelines for the care and use of animals.

### Data analysis

Data were analyzed with GraphPad Prism software. Nonlinear regression analyses were performed to generate dose-response curves and calculate IC_50_ values. Means ± SEMs were calculated automatically using this software. A paired two-tailed Student’s t-test was used to test for significance where indicated.

## Results

### Zardaverine selectively inhibits the growth and induces apoptosis of human HCC cells in vitro

To examine the antitumor activity of zadaverine, we first tested its anti-proliferative effect on a panel of solid human tumor cells, including liver, ovarian, lung, prostate, gastric, breast, glioblastoma, epidermoid and colon tumor cells. Zardaverine demonstrated potent antitumor activity against four HCC cell lines (Bel-7402, Bel-7404, QGY-7701 and SMMC-7721), with IC_50_ values ranging from 36.6 to 288.0 nM; however, it did not have any effect on the other fifteen cancer cell lines, including several HCC cell lines, with the IC_50_ values over 30 µM (Table 1). These results indicate that zardaverine has anti-proliferative effect and this effect is cell-type specific.

**Table 1:** Antitumor activity of zardaverine *in vitro*.

		IC_50_ (nM, Mean ± SEM)		
Tumor type	Cell line	Zardaverine	Vinorelbine	Paclitaxel
Liver	Bel-7402	137.7±9.6	5.5±0.2	0.9±0.1
	Bel-7404	288.0±59.8	37.1±2.0	5.3±0.7
	QGY-7701	51.0±1.8	5.3±0.3	0.9±0.1
	SMMC-7721	36.6±7.2	6.6±0.6	1.0±0.1
	Hep G2	>30,000	24.9±1.1	10.6±0.9
	Hep 3B	>30,000	39.0±0.8	21.3±1.0
	SNU-739	>30,000	6.0±0.3	1.1±0.1
	SNU-387	>30,000	45.0±6.6	1.5±0.5
	SNU-398	>30,000	7.4±1.4	8.8±2.0
	SNU-423	>30,000	19.5±2.4	0.9±0.2
	SNU-475	>30,000	39.0±1.8	19.2±1.3
Ovarian	SK-OV-3	>30,000	8.3±2.0	8.7±0.7
Lung	NCI-H460	>30,000	7.6±0.1	1.3±0.1
Prostate	LNCaP	>30,000	15.5±1.3	2.3±0.2
Breast	MDA-MB-231	>30,000	69.1±9.0	23.7±2.8
Gastric	NCI-N87	>30,000	11.4±0.6	6.9±0.3
Glioblastoma	U-87 MG	>30,000	13.6±1.0	15.5±1.8
Epidermoid	A431	>30,000	9.0±0.3	4.0±0.2
Colon	HCT 116	>30,000	26.7±1.2	7.2±0.2

Cells seeded in 96-well plates were treated with various concentrations of agents for 72 h and cell viability was determined by SRB assay. IC_50_ values represent the concentrations required to inhibit cell growth by 50%. All SRB data are presented as mean ± SEM from three independent experiments.

**Figure 1:**
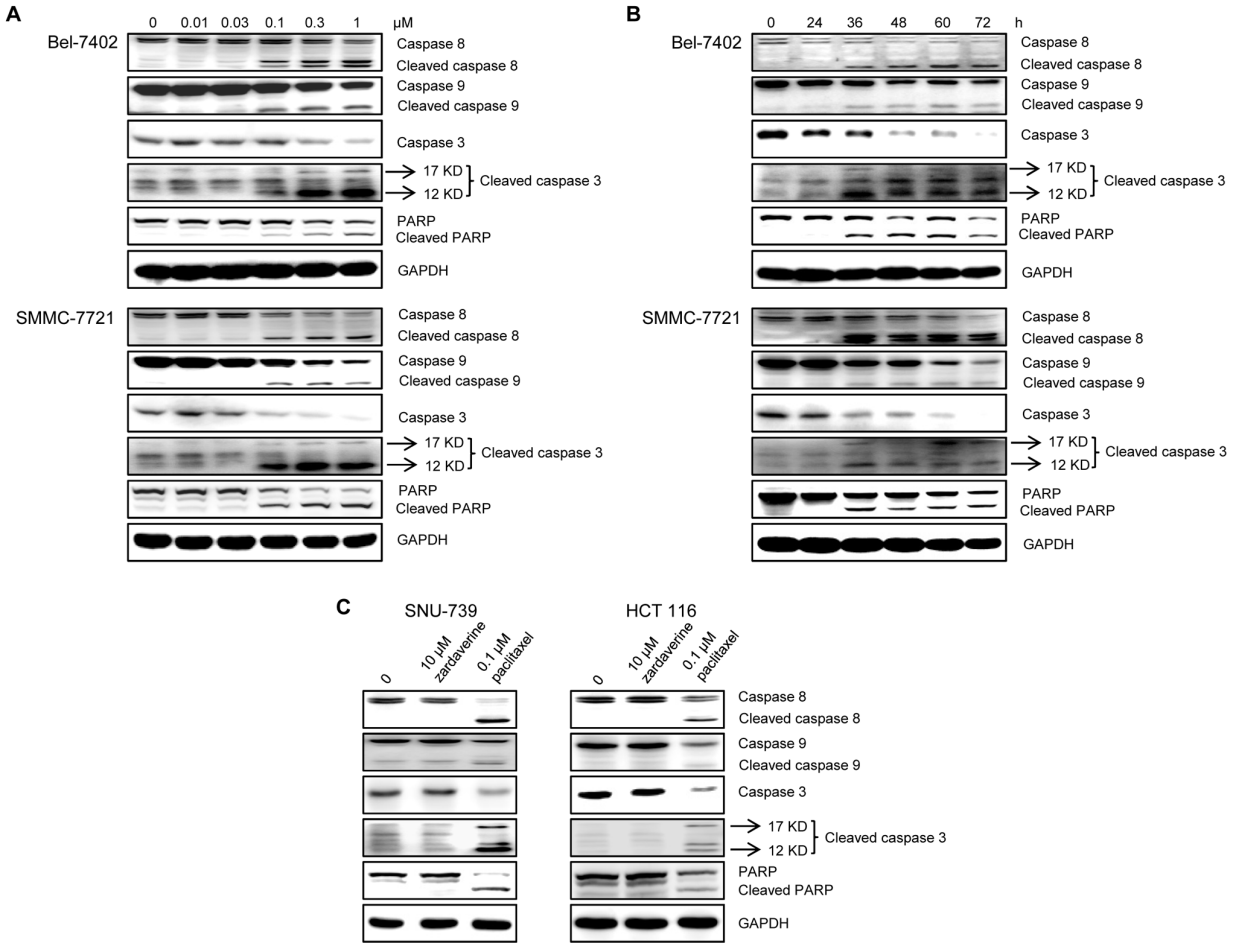
Zardaverine dose- and time-dependently induces apoptosis. **A**, Bel-7402 and SMMC-7721 cells were incubated with zardaverine for 48 h. **B**, Bel-7402 and SMMC-7721 cells were exposed respectively to 1 and 0.3 µM zardaverine for different periods. **C**, SNU-739 and HCT 116 cells were treated with 10 µM zardaverine for 72 h and 0.1 µM paclitaxel for 48 h. Whole-cell lysates were prepared and analyzed for cleavage of PARP, caspase-3, caspase-8, and caspase-9 by Western blotting.

We then investigated the effect of zardaverine on cell apoptosis by analyzing various cellular machinery mediators causing apoptosis. As shown in Fig. 1A, after 48 h of exposure, zardaverine induced a concentration-dependent increase in the cleavage of PARP and caspase-3, -8 and -9, which are apoptosis markers, in Bel-7402 and SMMC-7721 cells. In addition, when Bel-7402 and SMMC-7721 cells were exposed to 1 and 0.3 µM zardaverine respectively for different periods, apoptosis was induced in a time dependent manner (Fig. 1B). These results indicate that zadaverine dose- and time-dependently induces apoptosis in Bel-7402 and SMMC-7721 cells. In contrast, no apoptosis was observed in resistant SNU-739 and HCT 116 cells following 10 µM zardaverine treatment for 72 h (Fig. 1C). These results confirm the selectivity of antitumor activity of zardaverine.

### Zardaverine selectively inhibits the growth of human HCC xenografts in vivo

**Figure 2:**
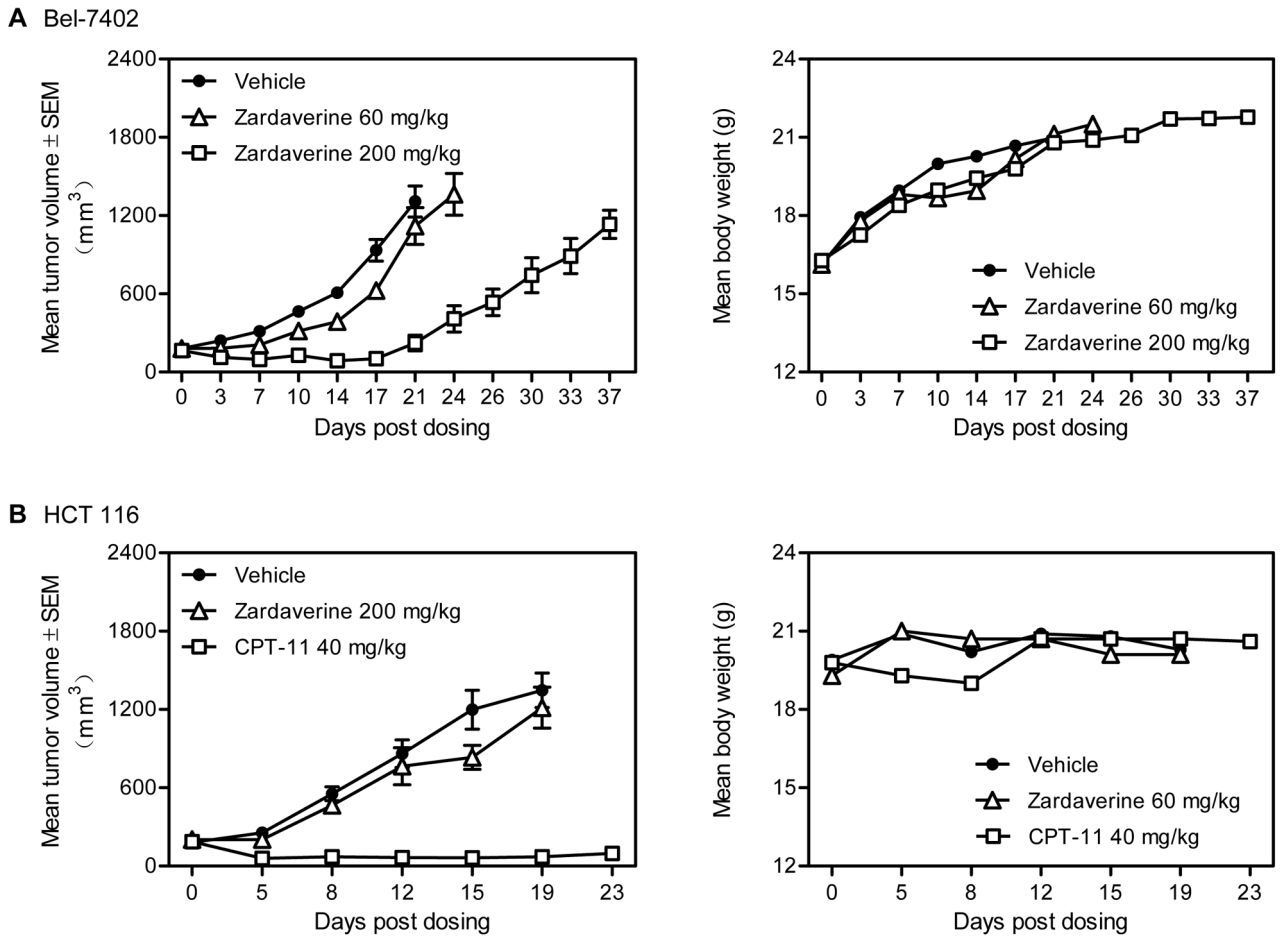
Zardaverine inhibits the growth of human tumor xenografts *in vivo*. A, Mice bearing human HCC Bel-7402 xenografts were orally administered zardaverine or vehicle daily for 14 days. **B,** Mice bearing human colon cancer cell HCT 116 xenografts were orally administered zardaverine or vehicle daily for 21 days and intraperitoneally administered CPT-11 every 4 days for three times. Tumor volume was measured on the indicated days. n  = 10 in vehicle group and n  =  6 in treatment groups.

Next, we evaluated the antitumor activity of zardaverine *in vivo.* As shown in Fig. 2A, zardaverine inhibited the growth of Bel-7402 xenografts at the dose of 60 mg/kg for 14 consecutive days and caused the tumor regression at the dose of 200 mg/kg. When zardaverine treatment was halted on the 14^th^ day, tumor regrew. Zardaverine treatment did not have any effect on the body weight of tumor-bearing mice and the tumor-bearing mice survived until the 37^th^ day when the experiment was halted (Fig. 2A). In contrast, zardaverine at the dose of 200 mg/kg had no effect on the growth of HCT 116 xenografts, in which reference drug CPT-11 caused the regression of tumors at the dose of 40 mg/kg (Fig. 2B). Collectively, these results are consistent with the *in vitro* results and indicate that zardaverine has significant and selective antitumor activity against certain HCCs both *in vitro* and *in vivo.*

### The selective antitumor activity of zardaverine is independent of PDE3/4 inhibition

Considering that zardaverine is a potent PDE3/4 inhibitor, we next investigated the relationship between the selective antitumor activity and PDE3/4 inhibition induced by zardaverine.

**Figure 3:**
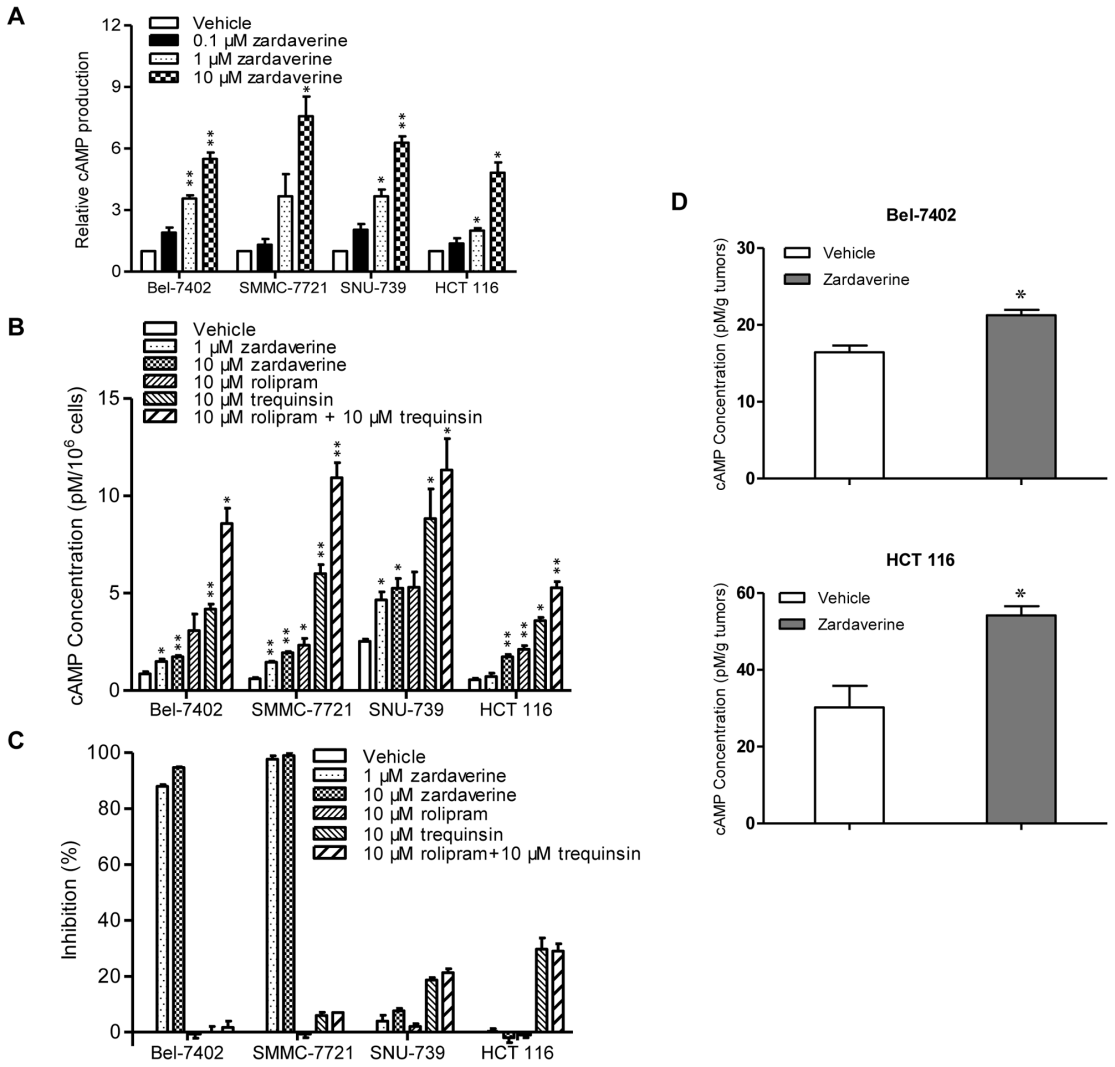
The selective antitumor activity of zardaverine is independent of PDE3/4 inhibition. **A,** Cells were incubated with 0.1, 1 and 10 µM zardaverine or vehicle for 1 h in the presence of 0.5 µM forskolin and intracellular cAMP levels were determined by a competitive immunoassay based on TR-FRET. RcP (relative cAMP production) was calculated from three independent experiments. **B,** Cells were incubated with different agents or vehicle for 24 h and intracellular cAMP levels were determined as noted in (**A**). Intracellular cAMP concentrations per 10^6^ cells (pM/10^6^ cells) were presented from three independent experiments. **C,** Cell proliferation inhibition effect of single or combined treatment with trequinsin and rolipram was determined by SRB assay. Proliferation inhibition rate of agents was compared to that of zardaverine in Bel-7402, SMMC-7721, SNU-739 and HCT 116 cells as shown in the histogram. **D**, Mice bearing human Bel-7402 and HCT 116 xenografts were orally administered zardaverine (200 mg/kg) or vehicle daily for 7 days, and cAMP levels of tumors at 3 h after the last administration were determined. cAMP concentration of tumors (pM/g ) was calculated as shown in the histogram. n  = 3 in both vehicle and treatment groups. Columns, means; bars, SEMs (n = 3; *p<0.05, **p<0.01).

To verify the inhibitory effect of zardaverine on PDE3/4, we first determined the ability of zardaverine to increase intracellular cAMP levels in the presence of forskolin which stimulates the synthesis of cAMP from ATP, using two zardaverine -sensitive cells Bel-7402 and SMMC-7721, and two zardaverine-resistant cells SNU-739 and HCT 116. To facilitate comparisons between cell types and experiments, we introduced the relative cAMP production (RcP), which is the ratio of the intracellular cAMP level of cells treated with zardaverine in the presence of forskolin to that of cells treated with foskolin only. The higher RcP means that higher level of intracellular cAMP is produced. As shown in Fig. 3A, zardaverine dose-dependently elevated the levels of intracellular cAMP of all four cells in the presence of forskolin. Zardaverine also slightly and dose-dependently increased the levels of intracellular cAMP of all four cells in the absence of forskolin (Fig. 3B). In addition, the single or combined treatment with trequinsin and rolipram, more potent PDE3 and PDE4 selective inhibitor [26], [27], [28], increased comparable cAMP levels in all tested four cells. These results indicate that all zardaverine, trequinsin and rolipram are potent PDE inhibitors capable to increase intracellular cAMP levels and there is no difference of PDE-cAMP pathways between zardaverine-sensitive and -resistant cells. It suggests that the selective antitumor effect of zardaverine may be independent of its PDE inhibition.

To test this hypothesis, we evaluated the anti-proliferative effect of 8-Br-cAMP, a cell permeable cAMP analog. 8-Br-cAMP dose-dependently inhibited cell proliferation of fourteen human tumor cells with inhibition rate ranging from 20% to 70% (data not shown). Importantly, the anti-proliferative effect of 8-Br-cAMP had no significant selectivity. It is speculated that the selective antitumor activity of zardaverine does not result from the up-regulation of intracellular cAMP by PDE3/4 inhibition.

To further confirm this hypothesis, we assessed the effects of trequinsin and rolipram on cell proliferation. Single or combined treatment of trequinsin and rolipram had no apparent growth inhibitory effect on both zardaverine-sensitive Bel-7402 and SMMC-7721 cells and zardaverine-resistant SNU-739 and HCT 116 cells (Fig. 3C). It was also the case for single or combined treatment with rolipram and amrinone, another PDE3 inhibitor [29] (data not shown). Considering that trequinsin and rolipram were more potent PDE3 and PDE4 inhibitor than zardaverine, these results clearly indicate that the selective antitumor activity of zardaverine is independent of PDE3/4 inhibition.

We also determined the cAMP levels of tumors from Bel-7402 and HCT 116 xenografts to confirm the effectiveness of zardaverine on PDE3/4 activity *in vivo*. As shown in Fig. 3D, the cAMP levels in both Bel-7402 and HCT 116 xenografts were significantly increased after mice were orally administered zardaverine for 7 days. These data not only confirm the *in vivo* inhibitory effect of zardaverine on PDE3/4, but also further confirm that the selective antitumor activity of zardaverine is independent of PDE3/4 inhibition.

### Zardaverine selectively causes G_0_/G_1_-phase arrest and dysregulates cell cycle-associated proteins in HCC cells

**Figure 4:**
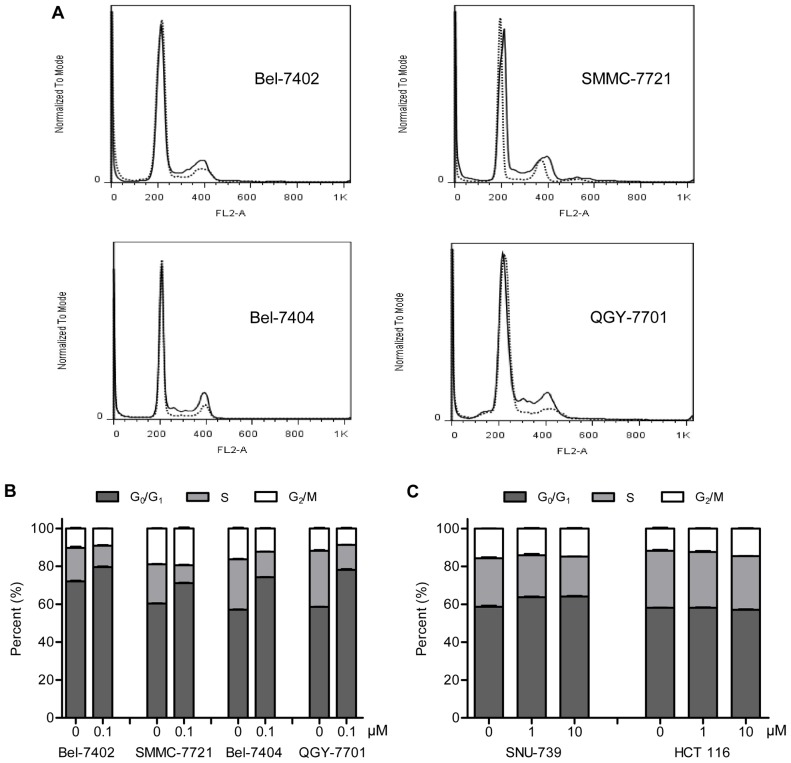
Zardaverine causes G_0_/G_1_ phase cell cycle arrest of sensitive cells. **A,** Bel-7402, SMMC-7721, Bel-7404 and QGY-7701 cells were treated with 0.1 µM zardaverine or vehicle for 24 h, then collected, stained with PI, and analyzed by flow cytometry. Representative flow histograms depicting cell cycle distribution are shown from three independent experiments with similar results. **—**, vehicle; 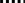, 0.1 µM zardaverine. **B,** The histograms from (**A**) were analyzed by the ModFit LT program, and the percentage of cells in each phase of the cell cycle is shown. **C,** SNU-739 and HCT 116 cells were treated with 1 and 10 µM zardaverine or vehicle for 24 h, then determined and analyzed as described above. Data shown in (**B**) and (**C**) are mean ± SEM from three independent experiments.

In order to understand the underlying mechanisms of selective inhibition of cell proliferation by zardaverine, we analyzed its effects on cell cycle phase distribution in the four sensitive (Bel-7402, Bel-7404, QGY-7701 and SMMC-7721) and two resistant (SNU-739 and HCT 116) cells. As shown in Fig. 4A and 4B, zardaverine induced accumulation of all four sensitive cells in the G_0_/G_1_ phase of the cell cycle at 0.1 µM concentration after 24 h treatment, and this accumulation of cells in G_0_/G_1_ phase caused by zardaverine was associated with parallel depletion of cells in the S phase. In contrast, zardaverine had no effect on the cell cycle phase distribution of resistant SNU-739 and HCT 116 cells even up to 10 µM (Fig. 4C). It was also the case for other several resistant cells (data not shown).

**Figure 5:**
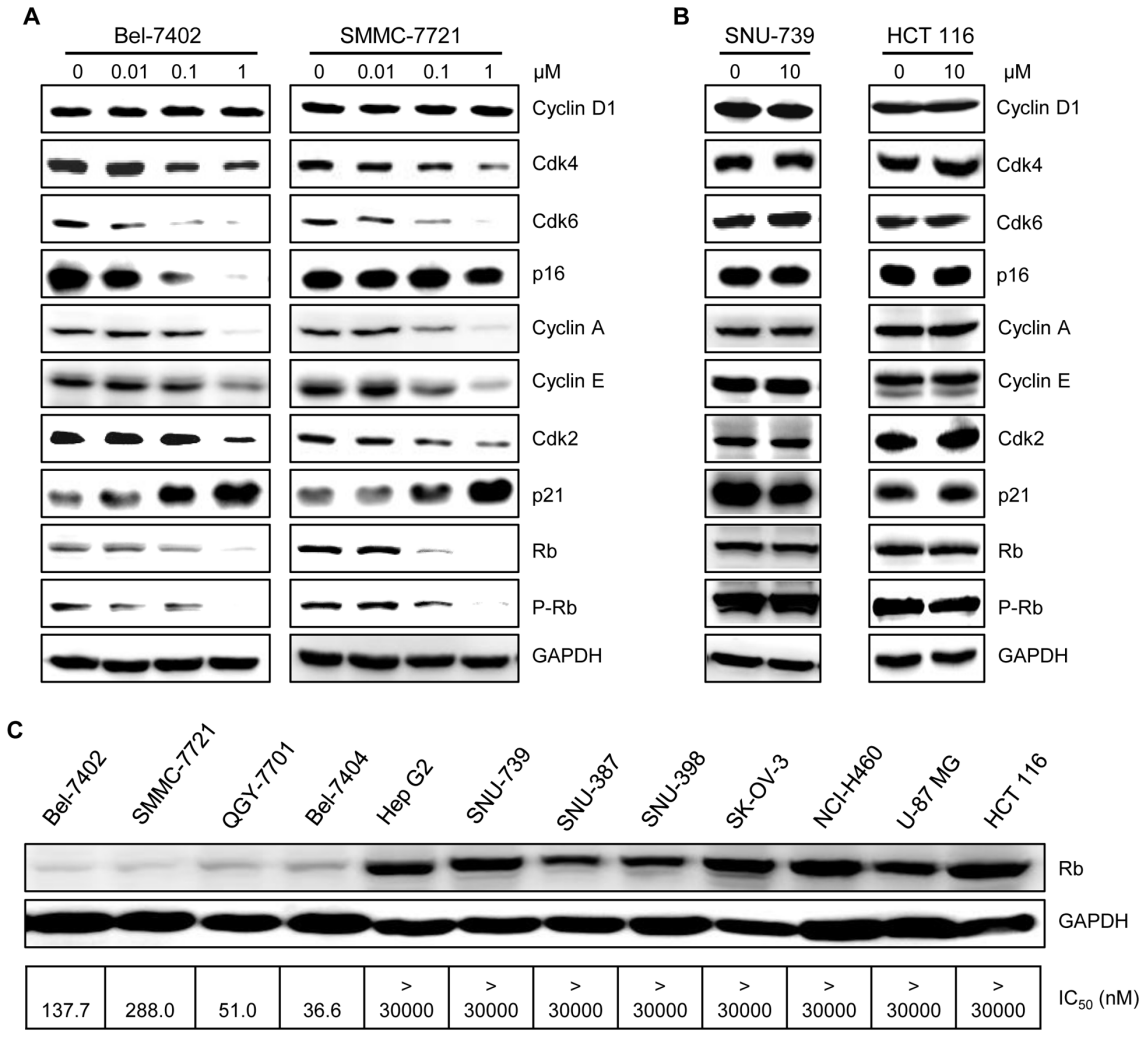
The selective antitumor activity of zardaverine is closely related to the regulation of cell cycle-associated proteins. Bel-7402 and SMMC-7721 cells (A), SNU-739 and HCT 116 cells (B) were incubated with zardaverine for 24 h. Then whole-cell lysates were prepared and analyzed by Western blotting. C, Cells were seeded in 6-well plates overnight. Whole-cell lysates were prepared, then total Rb levels were determined by Western blotting. IC_50_ values of zardaverine for these cells listed here are from Table 1.

To gain insights into the mechanism of zardaverine-induced G_0_/G_1_ phase cell cycle arrest, the expression of different proteins involved in regulating the G_1_ to S transition was monitored. Cell cycle progression is governed by cyclins and their kinase partners, the cyclin-dependent kinases (Cdks) [30]. Cdk4–Cyclin D and Cdk6–Cyclin D complexes regulate the G_0_–G_1_ transition, and Cdk2 is activated by E- and A-type cyclins during the G_1_ to S transition [31]. As shown in Fig. 5A, the protein levels of Cyclin A, Cyclin E, Cdk2, Cdk4 and Cdk6, but not Cyclin D1, were does-dependently suppressed by zardaverine in sensitive Bel-7402 and SMMC-7721 cells, whereas the protein level of the Cdk2 inhibitory subunit p21 was up-regulated. Interestingly, the Cdk4/6 inhibitory subunit p16 protein was reduced in a dose-dependent manner only in Bel-7402 cells, but not in SMMC-7721 cells (Fig. 5A), suggesting that p16 may be not essential for the antitumor activity of zardaverine. In resistant SNU-739 and HCT 116 cells, zardaverine did not cause any significant changes in the expression of Cyclin A, Cyclin E, Cyclin D1, Cdk2, Cdk4, Cdk6, p16 and p21 (Fig. 5B).

Rb, the product of the well-characterized tumor suppressor gene *Rb*, functions to prevent the cell from entering S phase, whereas phosphorylated or mutated forms of the protein are incapable of arresting the cell in G_1_ phase [32]. Zardaverine dose-dependently caused a decrease in protein level of total Rb and suppression of Rb phosphorylation at Ser780 in Bel-7402 and SMMC-7721 cells (Fig. 5A), while in SNU-739 and HCT 116 cells the levels of total Rb and phosphorylated Rb were not changed by 10 µM zardaverine exposure (Fig. 5B). More interestingly, the total Rb protein level of the zardaverine-sensitive cells was much lower than that of the resistant cells (Fig. 5C). The low level of Rb is closely related to the cell sensitivity to zardaverine. These findings collectively indicate that the selective antitumor activity of zardaverine is closely related to the regulation of cell cycle-associated proteins and that low expression of Rb is likely to be a biomarker for zardaverine-sensitive cancer cells.

## Discussion

Zardaverine, developed as a potential therapeutic agent for asthma because of its selective PDE3/4 inhibition in 1980s, has been reported to be a more potent inhibitor of PBMC and T-cell proliferation than rolipram. In this study, for the first time, we found that zardaveine exhibits significant and selective antitumor activity against HCC both *in vitro* and *in vivo*, and that this antitumor activity is independent of its PDE3/4 inhibition. We believe that zardaverine, or its structure-derivatives, has potential for further development, especially for the treatment of HCC.

Increasing intracellular levels of cAMP may cause growth arrest, induce apoptosis and attenuate migration in colon cancer cells and breast cancer cells [19], [33], [34], [35]. Hence, agents that raise levels of cAMP have gained attention as potential targets for anticancer therapy. Numerous studies provide evidence that PDE inhibitors may be effective agents for treating cancer. Here, we discovered that zardaverine exhibits selective antitumor activity against human HCC both *in vitro* and *in vivo*, however, its antitumor activity is independent of PDE3/4 inhibition, mainly based on the following facts: *a*, zardaverine effectively increases the intracellular cAMP levels *in vitro* and tumor cAMP levels *in vivo* without difference between zardaverine-sensitive and -resistant cells; *b*, the responses of zardaverine-sensitive and -resistant cells to 8-Br-cAMP are similar; *c*, the antitumor activity is not mimicked by trequinsin and rolipram, PDE3 and PDE4 inhibitor, albeit they could increase the levels of intracellular cAMP similar to or even more potent than zardaverine.

The fact that the antitumor activity of zardaverine is so specific to certain HCC cell lines urged us to focus on the genetic background of these cell lines. Most importantly but disappointingly, the genetic background of all the four sensitive cell lines (Bel-7402, Bel-7404, QGY-7701 and SMMC-7721) cannot be ascertained. Nevertheless, it is apparent that the selective antitumor efficacy of zardaverine is unrelated to the age, gender or ethnicity based on the clear genetic background of six resistant HCC cell lines (Hep G2: 15 years adolescent, male, Caucasian; Hep 3B: 8 years juvenile, male, Black; SNU-387: 41 years, female, Asian; SNU-398: 42 years, male, Asian; SNU-423: 40 years, male, Asian and SNU-475: 43 years, male, Asian) from ATCC. Additionally, hepatitis B virus (HBV) may not be involved in the selective anticancer effect of zardaverine because both HBV negative cell line Hep G2 and HBV positive cell lines (Hep 3B, SNU-387, SNU-398, SNU-423 and SNU-475) are resistant to zardaverine. Thus, further studies will be needed to gain insight into the mechanism mediating the selective antitumor activity of zardaverine.

Cell proliferation is well correlated to the regulation of cell cycle progression. To our excitement, zardaverine causes G_0_/G_1_ cell cycle arrest in sensitive cells and cell cycle-associated proteins including Cyclin A, Cyclin E, Cdk2, Cdk4, Cdk6 and p21 are dysregulated. Whereas, these observations are not found in resistant cells. Some of the molecules that control the early events of the cell cycle have been extensively characterized. The central players are the cyclin-dependent kinases (Cdks), a group of serine/threonine kinases that form active heterodimeric complexes following binding to cyclins [36]. Cdk4, Cdk6 and Cdk2 cooperate to drive cells through G1 into S phase. Cdk4 and Cdk6 are thought to be involved in early G1 whereas Cdk2 is required to complete G1 and initiate S phase. Cdk4 and Cdk6 form active complexes with the D-type cyclins while Cdk2 is sequentially activated by the E-type cyclins during the G_1_/S transition, and the A-type cyclins during S phase. Both Cdk4, Cdk6, Cdk2 and Cyclin A, Cyclin E decreased after zardaverine treatment in a dose-dependent manner, which provides evidence for G_0_/G_1_ phase arrest. However, the molecular mechanism by which these proteins were suppressed remains to be clarified. The Cdk inhibitor p21 plays an important role in the regulation of G_1_-S transition by binding to and inhibiting kinase activity of Cdk/cyclin complexes [37], [38]. Therefore, up-regulation of p21 also contributes to the G_0_/G_1_ phase arrest induced by zardaverine.

Rb, the product of the retinoblastoma-susceptibility gene *Rb*, was the first identified tumor suppressor protein [39]. Consistent with its tumor-suppressor function, Rb inhibits cell proliferation. The mechanism by which Rb inhibits cell growth has been elucidated. Through its interaction with the E2F family of transcription factors, Rb represses genes that are required for DNA synthesis [40]. However, the ability of Rb to suppress apoptosis is now supported by several lines of evidence [41], [42]. This apparent conundrum reveals an intriguing dual role of Rb: as an inhibitor of both cell growth and death. Rb is inactivated by phosphorylation following mitogenic stimulation, but it is degraded in response to death stimuli. Rb is an effector caspase substrate and caspase cleavage induces its degradation during apoptosis [43]. In present study, we demonstrate that in resistant cells, high expression of Rb protein were observed, however, extremely low Rb proteins were expressed in all sensitive HCC cells, and the protein levels of total Rb and Rb phosphorylation at Ser780 were significantly suppressed by zardaverine treatment. These results suggest that Rb may be a biomarker to evaluate the antitumor effect of zardaverine. Thus, to better understand the selective antitumor mechanism of zardaverine, we may focus on the mechanism of Rb downregulation or degradation, which may be an important clue to trace the exact target of zardaverine. Further investigation is underway.

The antitumor selectivity of zardaverine *in vivo* is consistent with that *in vitro*. More importantly, its antitumor activity *in vivo* is amazing, for it at the dose of 200 mg/kg induced Bel-7402 xenografts regression during the whole treatment without affecting mice weight. Though we have to sacrifice the mice in the vehicle-treated group bearing massive tumor on the 21^st^ day based on the animal ethics, the mice in the 200 mg/kg group treated with zardaverine for the first 14 days were still alive on day 37 with no symptoms of death, indicating that zardaverine significantly prolongs the lifetime of tumor-bearing mice. Thus, this amazing and potent antitumor activity of zardaverine *in vivo* plus Rb as a biomarker for zardaverine treatment prompt us to believe that zardaverine has potential for further development.

In summary, we show herein for the first time that zardaverine, a dual PDE3/4 inhibitor, exhibits potent and selective antitumor activity against HCC both *in vitro* and *in vivo* independently of PDE3/4 inhibition. We propose that low expression of Rb is likely to be a biomarker for cancer cells sensitive to zardaverine. These findings suggest that zardaverine or its analogs may have potential for the treatment of HCC, particularly for those expressing low level of Rb protein.
